# A critical review of brand and generic alendronate for the treatment of osteoporosis

**DOI:** 10.1186/2193-1801-2-550

**Published:** 2013-10-21

**Authors:** Jacques P Brown, Kenneth S Davison, Wojciech P Olszynski, Karen A Beattie, Jonathan D Adachi

**Affiliations:** Department of Medicine, Laval University, and CHU de Québec Research Centre, Quebec City, QC Canada; University of Victoria, Victoria, BC Canada; Department of Medicine, University of Saskatchewan and Saskatoon Osteoporosis Centre, Saskatoon, SK Canada; Department of Medicine, McMaster University, 501-25 Charlton Ave. East, Hamilton, ON L8N 1Y2 Canada

**Keywords:** Generic, Alendronate, Osteoporosis, Adverse event, Disintegration

## Abstract

**Objective:**

Compare *in vitro* and *in vivo* characteristics and clinical outcomes of brand and generic alendronate.

Research design and methods: Relevant search terms were input into Medline ("alendronate" AND "generic" up to August 5, 2013) and any abstracts deemed possibly relevant selected for full paper review and abstraction.

**Results:**

Multicentre, randomized, placebo-controlled Phase III clinical trials of substantial size and duration have established the anti-fracture efficacy and safety of brand amino-bisphosphonates. For regulatory approval, generic versions of brand drugs need to demonstrate bioequivalence in young, healthy volunteers and have similar dissolution times. While the potency and amount of active drug within generic formulations must be identical to the brand, differences are permitted in the excipients. Significant differences in tablet disintegration time among different versions of generic and brand alendronate have been reported. Rapidly disintegrating alendronate pills may increase oesophageal bioadhesion and adverse event risk. Oesophageal-bound alendronate or slow disintegrating alendronate tablets may be made inert and ineffective by subsequently ingested food or drink. Investigations have reported a lower persistence to therapy with generic brands of alendronate as compared to brand bisphosphonates and patients switched from brand to generic alendronate have increased adverse event rates and losses in bone mineral density.

**Conclusion:**

Numerous differences exist between brand and generic alendronate including: disintegration time, bioadhesion to the oesophagus, patient persistence to therapy, adverse event incidence, and maintenance of bone mineral density. Generic forms of alendronate warrant closer clinical study before they are ascribed the clinical effectiveness and tolerability of brand alendronate.

**Electronic supplementary material:**

The online version of this article (doi:10.1186/2193-1801-2-550) contains supplementary material, which is available to authorized users.

## Introduction

Osteoporosis is a systemic disease typified by decreased bone strength and a consequent increased risk of fragility fracture (2000). Fragility fractures are associated with significant decrements in quality of life (Papaioannou et al.^2008^) and decreased survival (Ioannidis et al.^2009^). Further, acute and chronic care for fragility fractures place a substantial economic burden on health care systems (Tarride et al.^2012^), which will only increase with the aging of the developed world’s population.

In light of the personal and societal costs of fragility fracture, their prevention is paramount. Positive lifestyle habits, such as varied physical activity and adequate vitamin D and calcium intakes, play an important role in the prevention of osteoporosis (Papaioannou et al.^2010^). However, once an individual has been found to possess a high risk for fracture (as assessed by a 10-yr fracture risk assessment tool), the clinical focus shifts towards treatment, which includes reinforcing positive lifestyle habits, but also necessitates the probable addition of pharmaceutical therapies to minimize this risk (Papaioannou et al.^2010^).

First-line therapy for the treatment of postmenopausal osteoporosis includes alendronate and risedronate (oral amino-bisphosphonates), zoledronic acid (intravenous amino-bisphosphonate), subcutaneous denosumab (RANK ligand inhibitor), raloxifene (selective estrogen receptor modulator), estrogen (hormone therapy) and teriparatide (recombinant parathyroid hormone) (Papaioannou et al.^2010^).

Of the first line therapies, the amino-bisphosphonates are the most widely used for the treatment of osteoporosis, demonstrating significant anti-fracture efficacy at vertebral, non-vertebral and hip sites when taken with concomitant calcium and vitamin D supplementation (MacLean et al.^2008^). Further, the safety and tolerability of the amino-bisphosphonates has been demonstrated in Phase III trials (Black et al.^1996^; Cummings et al.^1998^; McClung et al.^2001^; Harris et al.^1999^; Black et al.^2007^), with tens of thousands of women followed for a minimum three years. Continued efficacy and safety have been shown with alendronate out to ten years of administration (Bone et al.^2004^). Shortly after brand alendronate was released to market, there were numerous reports of gastrointestinal (GI) adverse events (AEs) with daily oral administration, particularly in the esophagus (Castell^1996^; de Groen et al.^1996^; Ettinger et al.^1998^). As a result of these reports, the alendronate tablet was reformulated with a waxy coating to minimize contact with the esophagus and strict dosing instructions were added to the package insert to ensure that the risk of exposure of alendronate to the esophageal mucosa was minimized. These changes to formulation and dosing, as well as later availability of weekly dosing, all but eliminated GI AEs with alendronate usage.

Despite the availability of proven anti-fracture medications, many patients who should be provided appropriate therapy do not receive it: the majority of women and men who suffer fragility fractures remain undiagnosed (>65%) and untreated (>70%) for osteoporosis (Giangregorio et al.^2006^; Bessette et al.^2008^). Of the relatively small proportion of patients who receive appropriate osteoporosis therapy, one of the most significant challenges is the ubiquitously poor adherence to therapy (Siris et al.^2009^; Imaz et al.^2009^). Both compliance (patient administers therapy at appropriate time and according to dosing instructions) (Weycker et al.^2007^) and persistence (how long a patient remains on the therapy) (Sheehy et al.2009a) are low for osteoporosis therapies, including the oral bisphosphonates. The anti-fracture benefits of osteoporosis medications increase with increasing compliance (non-linearly) (Siris et al.^2006^; Gallagher et al.^2008^; Rabenda et al.^2008^) and a minimum of six to twelve months of persistence is required in order to obtain the anti-fracture benefits of the amino-bisphosphonates (Black et al.^1996^; McClung et al.^2001^; Harris et al.^1999^; Black et al.^2007^). Since osteoporosis is asymptomatic until fracture, satisfactory adherence to therapy is even more difficult to obtain than with symptomatic diseases. The incidence of side-effects during osteoporosis therapy is a significant cause of non-adherence (Hansen et al.^2008^; Rossini et al.^2006^; Strampel et al.^2007^; Anastasilakis et al.^2007^) as patients may feel worse while taking therapy than when not. With adherence to therapy being disappointingly low with osteoporosis medications, the patient should be provided with a therapy that will minimize side-effects and maximize their ability to administer therapy accurately, reliably and for an extended period of time so that the anti-fracture benefits described in clinical trials can be realized.

The costs of health care are ever-escalating and as a consequence many public and private payees have mandated numerous measures to minimize expenditures. One of the most commonly-mandated cost-saving measures has been to enforce a program of systematic generic substitution for brand drugs after brand the patent has expired (Lai et al.^2012^; Grima et al.^2010^). The belief is that with this therapeutic substitution the patient will receive the same therapy, with all of its known benefits and risks, but at a lower cost to the payee. One study that followed a large cohort found that in the year 2008 81% of patients that started the year on brand alendronate had switched to generic alendronate by the end of the year (Yun et al.^2013^).

Alendronate was the first commercially-marketed amino-bisphosphonate for the treatment of osteoporosis and, consequently, the first to lose its patent and be provided to the market as a generic drug. Recently, risedronate (5 mg/d and 35 mg/w doses) has also been made available as a generic option in a number of countries around the world. After its introduction, generic alendronate was widely adopted by public and private payees owing to its lower price as compared to brand alendronate.

This review has the objective of detailing all published *in vitro, in vivo*, clinical outcome and database comparisons between brand and generic alendronate to establish whether the adherence, tolerance and efficacy results obtained from Phase III clinical trials with brand alendronate can be reasonably ascribed to generic forms of alendronate. Further, a review of the cost-effectiveness data is made.

## Methods

Medline was searched to identify potential papers for inclusion for this review, keywords "alendronate" AND "generic" with the limitations of papers published between January 1, 1995 and Aug 5, 2013 published in English (n = 51 papers). After reviewing each reference’s title and abstract, all papers that were deemed possibly relevant to the topic were retained and had the full paper acquired (n = 32). Reasons for non-inclusion (19) were: non-english language (1), review (5), commentary (3), not primary focus (6) and position statements (4).

Reference lists from acquired papers were scanned for possible new citations. Numerous papers not gathered from the search were included to provide perspective to the discussion.

## Results

When a patient chooses generic alendronate, or has it chosen for them through mandatory substitution (Strom & Landfeldt^2012^), there is an underlying assumption that the tolerability, safety and effectiveness of the generic version of alendronate are equal to that of brand alendronate.

Several federal guidelines exist to provide direction to companies that manufacture and market generic versions of a branded drug (Federal Drug Administration^2008^; Health Canada^1997^). Generic versions must contain identical amounts of the active ingredient(s) in an identical dose formulation and the same route of administration as the brand and must adhere to rigid guidelines with respect to allowable limits for active drug strength, purity and quality. However, variations between the brand and generic version in the inactive ingredients (excipients), such as binders, fillers and disintegrants, are permitted so long as the excipients exist in a similar ratio to that of the excipients in the brand formulation (Meredith^2003^).

Differences in excipient composition and behaviour may result in altered disintegration or dissolution times which could, in turn, change the bioavailability, pharmacokinetics and tolerability of the active drug. If differences in generic tablet composition exist, the tolerability, safety and efficacy of a brand therapy reported from clinical trials should not be automatically ascribed to the generic version of a drug until otherwise confirmed by assessing the generic in similarly designed randomized control trials. Unfortunately, these confirmatory trials are rarely, if ever, performed before regulatory permissions are given for the marketing and sale of generics.

### Bioavailability

Bioavailability is the degree of activity or the amount of an administered drug or other substance that is made available for action on the target cells. Changing a drug’s bioavailability can lead to alterations in its safety and/or efficacy profiles. The bioavailability of alendronate is exceedingly low (0.64%) when given on an empty stomach to healthy, young volunteers under ideal circumstances (Prescribing information^2010^). If alendronate is taken with food or liquids other than water it is made unavailable to the body due to binding with the food or drink and passing through the digestive tract unabsorbed (Porras et al.^1999^). Consequently, it is recommended that the drug be taken first thing in the morning with water before ingesting any food or beverages with subsequent food and beverages withheld for at least 30 minutes following the administration of alendronate. Further, the patient should remain upright for at least 30 minutes after ingestion to minimize the risk of GI reflux and oesophageal irritation (Perkins et al.^2008^).

Differences in the excipient composition between the brand and generic formulations of alendronate may alter the bioavailability of the generic alendronate to bone if this substitution changes the behaviour of the tablet such that the alendronate tablet becomes more easily bound to food or drink and unavailable for absorption in the gut.

### Bioequivalence

Bioequivalence is defined by the Federal Drug Administration of the United States as "the absence of a significant difference in the rate and extent to which the active ingredient or moiety in pharmaceutical equivalents or pharmaceutical alternatives becomes available at the site of drug action when they are administered at the same molar dosage under similar conditions in an appropriately designed study" (Federal Drug Administration^2003^).

Generic versions of alendronate have been reported to be bioequivalent to branded alendronate (Rhim et al.^2009^; Yun et al.^2006^; Lainesse et al.^2004^). The development of a brand name formulation requires the demonstration of its pharmacokinetics, efficacy and tolerability in both healthy subjects and in the target patient population. However, the development of the generic equivalent requires only the demonstration of its bioequivalence with the brand name product in healthy subjects (Dighe^1999^). The World Health Organization guidelines state that 18 to 24 healthy male and female volunteers aged 18 to 55 years of normal body weight should be used in a crossover study design to determine whether bioequivalence is achieved between two formulations (World Health Organization^1996^). It is assumed that bioequivalence demonstrated in crossover studies performed in this typically younger, healthier population would be equivalent to that observed in the patient population. It is further supposed that this bioequivalence would translate into comparable clinical efficacy and tolerance; however, evidence to support the existence of a well-defined relationship between these parameters is lacking (Meredith^2003^). These differences have fostered concern as to whether bioequivalence, as ascertained in crossover studies of healthy adults, should be used to make claims of comparable clinical effectiveness and tolerability in patients who are older and often have numerous underlying disorders or diseases. Further, older individuals tend to have greater difficulty in swallowing pills (orientation of pill in mouth) and have reduced GI motility as compared to younger individuals. These differences may increase the exposure time of the alendronate tablet to the upper GI tract which could then increase the probability of alendronate exposure to the esophagus in older adults.

### *In vitro* and *in vivo* comparisons of brand and generic alendronate

A number of *in vitro* investigations have compared pertinent physical characteristics of generic alendronate and brand alendronate, with the majority finding notable differences.

#### *In vitro*: disintegration and dissolution

Since the bioavailability of alendronate is low and the dosing requirements strict, any characteristics of tablets that change the speed of delivery of alendronate, such as time required for disintegration or dissolution, could have important implications on drug effectiveness or tolerance. For regulatory approval, there are explicit dissolution parameters required to allow for the generic substitution of brand alendronate, however there are no specifications as to the required disintegration characteristics of the generic forms.

Disintegration is the physical process where the tablet breaks down into fine particles. It is monitored visually and relates to the physical integrity of the tablet. It is important to note that disintegration assays are not sufficient to establish absorption rates – disintegration is the initial step in drug release and its determination is important as a potential limiting factor in overall drug release (Bolanos^2004^). Dissolution is the process by which the active ingredient is dissolved into the liquid assay medium. Dissolution is assessed via chemical analysis and provides the approximate time required for full solubilisation of the drug under test conditions.

With rapid drug disintegration there is a greater chance of adhesion of the tablet to the oesophageal mucosa, thereby increasing the risk of irritation and ulceration. Further, rapidly-disintegrating alendronate that is adhered to the oesophagus may subsequently become inert after contact with food or drink. In contrast, very slow disintegration may increase the probability of the drug coming into contact with subsequently ingested food of drink, binding the alendronate and negating any possible positive anti-fracture benefit.

Table 1 presents disintegration comparisons of brand and generic forms of alendronate from all available trials. Epstein et al. (2003) compared the disintegration profile of brand alendronate with that of 13 generic versions of alendronate available in Latin America. There were a large number of generic versions of alendronate that disintegrated faster than brand alendronate (brand alendronate mean disintegration time of 1.4 minutes; generic mean disintegration times ranging from 6.9 to 46.5 seconds) and generic versions that took far longer than brand alendronate to disintegrate (mean disintegration time ranging from 10.3 to 46.5 minutes).

**Table 1:** **Disintegration rates of brand alendronate and generic alendronate**

Lead author, year	Brand or generic	Dose	Trade or Manufacturer name	Country of origin	n	Dsnt time (s) (mean)	Dsnt time (s) (sd)	% RSD	Dsnt < 30s
**Epstein, 2003**	Brand	70mg	Fosamax, Merck & Co., Inc.	US	27	86.1	11.8	14%	N
	Generic	70mg	Fosval	Chile	12	6.9	1.5	23%	Y
	Generic	70mg	Osteoplus	Ecuador	12	14.7	1.6	11%	Y
	Generic	70mg	Fixopan	Ecuador	14	16.2	2.1	13%	Y
	Generic	70mg	Osteomix	Ecuador	12	22.4	2.1	9%	Y
	Generic	70mg	Genalmen	Venezuela	8	26.1	1.9	7%	Y
	Generic	70mg	Genalmen	Venezuela	4	19.5	3.1	16%	Y
	Generic	70mg	Endronax	Brazil	12	32.8	4.7	15%	N
	Generic	70mg	Osteomax	Costa Rica	13	44.2	3.7	8%	N
	Generic	70mg	Fosmin	Peru	10	46.5	11.6	25%	N
	Generic	70mg	Defixal	Venezuela	12	46.5	4.4	10%	N
	Generic	70mg	Ostenan	Brazil	14	25.0	11.2	45%	Y
	Generic	70mg	Regenesis	Argentina	20	13.0	1.7	13%	Y
	Generic	70mg	Neobon	Columbia	23	13.0	6.7	52%	Y
**Dansereau, 2008**	Brand	70mg	Fosamax, Merck & Co., Inc. "A"	US	4-6	43	11	26%	N
	Brand	70mg	Fosamax, Merck & Co., Inc. "B"	US	4-6	78	12	15%	N
	Brand	70mg	Fosamax, Merck & Co., Inc. "C"	US	4-6	52	23	44%	N
	Brand	35mg	Actonel, P&G Pharmaceuticals "A"	US	4-6	35	3	9%	N
	Brand	35mg	Actonel, P&G Pharmaceuticals "B"	US	4-6	44	7	16%	N
	Brand	35mg	Actonel, P&G Pharmaceuticals "C"	US	4-6	49	6	12%	N
	Generic	70mg	Apotex "A"	Canada	4-6	198	78	39%	N
	Generic	70mg	Apotex "B"	Canada	4-6	132	48	36%	N
	Generic	70mg	Apotex "C"	Canada	4-6	192	48	25%	N
	Generic	70mg	Cobalt "A"	Canada	4-6	150	42	28%	N
	Generic	70mg	Cobalt "B"	Canada	4-6	174	48	28%	N
	Generic	70mg	Novopharm "A"	Canada	4-6	21	7	33%	Y
	Generic	70mg	Novopharm "B"	Canada	4-6	13	1	8%	Y
	Generic	70mg	Novopharm "C"	Canada	4-6	24	50	208%	Y
	Generic	70mg	Pharmascience	Canada	4-6	126	48	38%	N
**Dansereau, 2009**	Brand	70mg	Fosamax, Merck & Co., Inc.	US	6	53	9	17%	N
	Generic	70mg	Teva Pharma "A"	US	6	60	24	40%	N
	Generic	70mg	Teva Pharma "B"	US	6	27	3	11%	Y
	Generic	70mg	Teva Pharma "C"	US	6	72	6	8%	N
	Generic*	70mg	Watson Pharma	US	6	108	42	39%	N
	Generic	70mg	Barr Laboratories, Inc "A"	US	6	10	2	20%	Y
	Generic	70mg	Barr Laboratories, Inc "B"	US	6	9	1	11%	Y
	Generic	70mg	Barr Laboratories, Inc "C"	US	6	9	2	22%	Y
	Generic	70mg	Aluid	Germany	4-6	84	18	21%	N
	Generic	70mg	AWD	Germany	4-6	168	18	11%	N
	Generic	70mg	Betapharm "A"	Germany	4-6	342	96	28%	N
	Generic	70mg	Betapharm "B"	Germany	4-6	156	42	27%	N
	Generic	70mg	Betapharm "C"	Germany	4-6	174	48	28%	N
	Generic	70mg	GRY	Germany	4-6	21	5	24%	Y
	Generic	70mg	Hexal "A"	Germany	4-6	336	66	20%	N
	Generic	70mg	Hexal "B"	Germany	4-6	60	6	10%	N
	Generic	70mg	Hexal "C"	Germany	4-6	150	54	36%	N
	Generic	70mg	Heuman	Germany	4-6	168	24	14%	N
	Generic	70mg	Juta	Germany	4-6	30	4	13%	N
	Brand	70mg	Fosamax, Merck & Co., Inc.	Germany	4-6	84	18	21%	N
	Generic	70mg	Ratiopharm "A"	Germany	4-6	84	24	29%	N
	Generic	70mg	Ratiopharm "B"	Germany	4-6	246	42	17%	N
	Generic	70mg	Ratiopharm "C"	Germany	4-6	198	36	18%	N
	Generic	70mg	Stada "A"	Germany	4-6	186	84	45%	N
	Generic	70mg	Stada "B"	Germany	4-6	186	96	52%	N
	Generic	70mg	Stada "C"	Germany	4-6	222	24	11%	N
	Generic	70mg	Apothecon	Netherlands	4-6	106	30	28%	N
	Generic	70mg	Kromme	Netherlands	4-6	58	14	24%	N
	Generic	70mg	Centrapharm	Netherlands	4-6	330	60	18%	N
	Generic	70mg	Pharmachemie	Netherlands	4-6	14	4	29%	Y
	Generic	70mg	Ratiopharm	Netherlands	4-6	66	18	27%	N
	Generic	70mg	Sandoz	Netherlands	4-6	72	30	42%	N
	Generic	70mg	APS/Teva "A"	UK	4-6	37	9	24%	N
	Generic	70mg	APS/Teva "B"	UK	4-6	26	5	19%	Y
	Generic	70mg	Arrow "A"	UK	4-6	144	60	42%	N
	Generic	70mg	Arrow "B"	UK	4-6	34	9	26%	N
	Generic	70mg	Pliva	UK	4-6	60	12	20%	N
	Generic	70mg	Ratiopharm	UK	4-6	78	18	23%	N
	Generic	70mg	Teva "A"	UK	4-6	14	3	21%	Y
	Generic	70mg	Teva "B"	UK	4-6	29	5	17%	Y
	Generic	70mg	Winthrop "A"	UK	4-6	132	36	27%	N
	Generic	70mg	Winthrop "B"	UK	4-6	306	36	12%	N

*manufactured by Merck, sold by Watson Pharma Inc.

%RSD = relative percent standard deviation; n = number of tablets tested; Dsnt = disintegration in seconds.

In a study by Dansereau et al. (2008), the dissolution and disintegration of a number of generic formulations of alendronate from Canada, the Netherlands, Germany, and the United Kingdom, were compared to United States-manufactured brand risedronate and alendronate. All of the generics tested had an acceptable dissolution rate as compared to brand alendronate. Commercially-available orally disintegrating tablets designed to dissolve in the mouth without water prior to swallowing were purposefully included to act as disintegration comparators. Six of the 26 generic versions of alendronate tested had a disintegration time that was comparable to that of the orally-disintegrating tablets. Similarly, in a later study by Dansereau et al. (2009), the majority of US-based generics tested disintegrated rapidly enough to meet the regulatory guidelines for an orally-disintegrating tablet.

In contrast to the above data, an investigation from Portugal reported that there were no significant differences between the generic and brand alendronate formulations for either dissolution or disintegration parameters (Almeida et al.^2006^). Another trial reported that brand alendronate was more likely to cause GI injury than generic versions since the generic brands released significantly lower amounts of alendronate into solution than the original product (Lamprecht^2009^).

The majority of generic versions of alendronate disintegrate faster or slower than brand alendronate, whereas dissolution times are largely similar between brand and generic alendronate.

#### *In vivo*: cleavage rupture and oesophageal adhesion

GI side effects are one of the most commonly reported side effects associated with amino-bisphosphonates (Black et al.^1996^; Cummings et al.^1998^; Ettinger et al.^1998^). Alendronate-associated GI AEs have important consequences, not only for patients’ health but also for inducing GI-related direct and indirect medical costs (Kane et al.^2004^).

Pill-induced oesophagitis occurs primarily when the ingested tablet adheres to the epithelial surface of the oesophagus after swallowing and lesions develop. The potential for developing pill-induced oesophagitis is dependent on a number of factors including patient age-related impairment of oesophageal motility, local pH, the amount of water administrated with the pill, patient position and formulation characteristics, including size, shape and coating (Drake et al.^2002^). Alendronate has a high degree of oesophageal toxicity, owing to its very low pH in aqueous solution (de Groen et al.^1996^; Epstein et al.^2005^; Dobrucali et al.^2002^) and if gastric acid is refluxed after the administration of alendronate the local damaging effect of alendronate may be enhanced in this now lower pH environment (Dobrucali et al.^2002^). It is recommended to take a full glass of water after swallowing the alendronate pill to ensure that is clears the oesophagus rapidly. Oesophageal irritation, and even ulceration, have been reported in patients who were non-complaint in the dosing instructions to take the therapy with sufficient water and to avoid lying down or being semi-supine for a period of half an hour after administration (Castell^1996^; de Groen et al.^1996^; Strampel et al.^2007^; Naylor & Davies^1996^). However, there have been reports of oesophageal irritation even in those who claim compliance to the dosing instructions.

Further, clinical concentrations of both alendronate and risedronate have been demonstrated to suppress the growth of epidermal keratinocytes through the same mechanisms that are integral in their control of osteoclast activity – inhibition of farnesyl diphosphate synthase (Reszka et al.^2001^). A few trials have suggested that the combination of alendronate and non-steroidal anti-inflammatory medications may have a synergistic ulcerogenic effect (Ettinger et al.^1998^; Graham & Malaty^2001^).

A number of investigations have studied the impact of different excipients on the rupture characteristics between generic and brand alendronate tablets and whether this plays a role in oesophageal adhesion.

If the alendronate tablet is ingested with little or no water it can come into direct contact with the oesophageal epithelium and can even become compressed against the esophagus during the contraction phase of peristalsis during swallowing (Dai et al.^2003^; Mittal et al.^2005^). It is possible during this compression and in the absence of sufficient water for bioadhesion forces created from the solid form to be greater than the detachment forces produced from drinking liquids or from subsequent peristaltic movements.

Shakweh et al. (2007) investigated the bioadhesive characteristics of generic alendronate manufactured in Europe and brand alendronate, along with one negative and two positive polymer controls, in a porcine oesophageal model. The brand alendronate had little to no bioadhesive characteristics whereas the generic tablets made by Teva had bioadhesive characteristics that were similar to the positive control. Several other forms of generic tablets (Alenat, Stada, Aliud, Ratiopharm) displayed cleavage rupture in the oesophagus leaving a large piece of tablet strongly adhering to the mucosa. The brand alendronate studied did not demonstrate this rupturing behaviour. In cleavage rupture the adhesive forces from the mucosa were greater than that of the tablet cohesiveness. It is possible in this circumstance that drinking sufficient water when taking the tablet would force it down the oesophagus without adhesion to the oesophageal wall. However, if insufficient water was consumed or the adhesive strength was too great, adhesion may occur resulting in irritation of the mucosa. It was hypothesized that the differences in bioadhesion and cleavage rupture were likely due to differing inactive ingredients.

Perkins et al. (2008) assessed the oesophageal transit time of branded risedronate and two generic alendronate formulations. It was found that a semi-sitting posture, as opposed to being upright, significantly (p < 0.05) slowed oesophageal transit time. Further, the generic alendronate formulations had significantly (p < 0.01) slower transit times than the risedronate tablets. Further, rupture of generic alendronate tablets was noted in some of the trials, in both the upright and semi-supine positions. Slower transit times in combination with rapid disintegration of generic tablets could greatly increase the likelihood of mucosa adhesion and exposure.

Epstein et al. (2005) used a canine model to assess differences in oesophageal tolerability between brand and generic alendronate. In the study the dogs were exposed to generic or brand alendronate on the caudal third of the oesophagus for one hour in each of five consecutive days (saline rinse after each application). All four of the dogs that were exposed to the generic alendronate exhibited marked ulcerative oesophagitis whereas it only developed in one for five of the dogs that were exposed to brand alendronate.

In conclusion, laboratory data demonstrates that there are frequent differences between brand and generic alendronate in GI transit time, in bioadhesion to the oesophagus and in the propensity for tablet rupture.

### Clinical data

In clinical trials, brand alendronate has similar safety and tolerability profiles as compared to placebo (Black et al.^1996^; Cummings et al.^1998^; Liberman et al.^1995^; Devogelaer et al.^1996^; Tonino et al.^2000^; Schnitzer et al.^2000^; Rizzoli et al.^2002^). Therefore, any differences in tolerability in the real world could potentially be due to either improper dosing or to the different formulations of alendronate.

When investigating whether there are differences in safety or effectiveness between generic versions of alendronate and the brand version, a common approach is to assess experiences in clinical populations outside of the trial setting. Chart reviews of community-based patients can provide real-world data comparing important outcomes with respect to tolerance and effectiveness of brand and generic versions of alendronate.

In a Canadian study, a chart review of 301 women from two specialized tertiary care referral centers was undertaken to quantify changes in AE rates, changes in bone mineral density, and discontinuation among postmenopausal women greater than 50 years of age before and after switching from brand to generic alendronate (Grima et al.^2010^). Patients who were previously stable on doses of brand alendronate experienced an increase in AEs which were serious enough to result in discontinuation after introduction of automatic substitution to generic alendronate. In addition, reductions in bone mineral density were observed in some patients who had stable bone mineral density while on brand alendronate (prior to January 2005). Given the substantial increase in AEs recorded after the automatic substitution of brand alendronate by generic alendronate it could be suggested that generic alendronate may not be as well tolerated as brand alendronate.

In another chart review, this time of a German practice, Ringe and Moller (2009) reported differences in persistence, safety and effectiveness in 186 postmenopausal women with osteoporosis treated with brand alendronate, brand risedronate or generic alendronate over a period of a year. After a year of therapy, the mean increases in bone mineral density at both the lumbar spine and total hip were significantly lower (p < 0.05) for the generic alendronate group as compared to either of the groups of women provided brand bisphosphonates. After a year, only 68% of the patients taking generic alendronate were persistent with therapy, as compared to 84% of the patients taking brand alendronate and 94% of the patients taking brand risedronate (p < 0.05 between generic vs either brand). Perhaps explaining the lower persistence with generic alendronate was the significantly (p < 0.05) higher rate of AEs in the women taking generic alendronate (n = 32) as compared to those women taken brand alendronate (n = 15) or brand risedronate (n = 9).

Lai et al. (2012) assessed the impact of mandatory generic alendronate substitution on adherence and adverse event incidence. In this small investigation, there were no significant differences between generic and brand alendronate with respect to medication adherence, but patients that were provided generic alendronate had a significantly greater incidence of AEs as compared to those women on the brand formulation (OR = 7.84; 95% CI: 2.98, 20.65).

Thus, real-world clinical data demonstrates that when generic alendronate is compared to brand alendronate there are significant differences in persistence to therapy, effectiveness (bone mineral density) and safety (AEs).

### Database analyses

Another common approach to compare whether there are differences in persistence, safety or effectiveness between generic versions of alendronate and the brand version is to perform analyses of administrative databases.

In an administrative database analysis, information regarding persistence to therapy of once-weekly bisphosphonates was obtained from a large cohort of patients by linking the administrative databases of the Régie de l’Assurance Maladie du Québec (RAMQ) (Sheehy et al.2009a). The RAMQ database collects prescribing and prescription filling information for all residents of the province of Quebec, Canada who were not covered by private health insurance (60% of residents aged 18–64 and almost 98% of 65 and older) (Régie de l'assurance maladie du Québec^2010^).

In this analysis, data was isolated for bisphosphonate-naïve patients who were started on brand risedronate (13,441 patients), brand alendronate (18,488 patients), or generic alendronate (875 patients) and the patients were then followed forward in time for a year from their first prescription. Patients who were initiated on weekly generic alendronate had a significantly (p < 0.0001) lower persistence to therapy as compared to those patients who began weekly brand risedronate or brand alendronate therapy. Even after adjusting for confounding covariates (female gender, socioeconomic status, previous osteoporotic fracture, number of family physician or specialist visits in past year, number of days in hospital in the past year, number of days in the ER in past year and chronic disease score), patients initiated on weekly generic alendronate had a significantly higher probability of discontinuing therapy as compared to either brand risedronate or alendronate (Hazard ratio = 2.08, 95% CI: 1.89-2.28). The significant difference between persistence to the generic versus the brand bisphosphonates may be attributable to physiochemical differences between the formulations, or to differences in the populations dispensed the drugs.

Another investigation using the same administrative RAMQ databases as the study above concluded that persistence to brand bisphosphonates was significantly (p < 0.001) higher than to generic alendronate for patients who either had (primary prevention) or had not (secondary prevention) previously experienced an osteoporotic fracture (Sheehy et al.2009b). Even after controlling for numerous covariates there was significant risk for discontinuation with generic alendronate as compared to brand alendronate (hazard ratio = 2.08, 95% confidence interval 1.89–2.28).

Strom and Landfeldt (2012) conducted an analysis of data from a large Swedish database (Swedish Prescribed Drug Registry) that contained information of prescription dispensation from 2006 through 2009. They reported that automatic generic substitution of generic alendronate was associated with reduced treatment persistence. From 2006 to the end of 2009 automatic substitution of generic alendronate increased from 11% to 45% with a concomitant decrease in one-year persistence to therapy from 67% to 52%. Those patients that had their alendronate substituted at their first prescription refill had a significantly higher probability of discontinuing their therapy (HR = 1.25; 95% CI: 1.2, 1.3).

In contrast to the above investigations, van Boven et al. (2013) found that use of generic alendronate was not associated with a decreased one-year persistence to therapy as compared to other brand bisphosphonates. Specifically, no difference was observed between the branded and generic forms of alendronate (HR = 1.00; 95% CI: 0.89, 1.12).

In 2005, the patent for alendronate expired in the UK and there was large-scale switching of bisphosphonate users to the generic formulation of alendronate. Ralston et al. (2010) conducted a database analysis (retrospective cohort study) to investigate whether there was a difference in GI tract AEs between patients who were provided risedronate and remained on risedronate and those who began on risedronate and switched to alendronate after March 2005 (generic introduction). The investigators found that there was an increased risk of GI AEs for those patients switched from risedronate to alendronate (HR = 1.85; CI: 1.26, 2.72), with a greater risk in those with a history of GI events (HR = 3.18; 95% CI: 2.79, 3.63).

In a retrospective database health resource utilization analysis of upper GI tract outcomes in patients started on either generic (from Israel) or brand alendronate, it was concluded that there were significantly higher discontinuation rates with the generic versions of alendronate as compared to the brand alendronate (incident rate ratios 1.3-1.59; p < 0.05) (Halkin et al.^2007^). However, in this investigation the authors found no difference between users of brand and generic alendronate in the incidence of upper GI AEs. Specifically, there were no differences between brand and generic alendronate for visits to a gastroenterologist (<1%), for GI-related hospitalizations (2.9-3.2%) and for rates of new gastric drug therapy (3.5– 4.9%). Further, for those who required an endoscopy investigations there were no differences in endoscopic diagnoses among the formulations tested. Notably, the authors excluded any patient with prior upper GI tract problems or patients that discontinued in the first three months. Different from the other investigations (Sheehy et al.2009b; Blouin et al.^2009^) was the fact that the database only followed the first 90–120 days of alendronate prescription; it may be that the AEs associated with generic versions of alendronate become more evident after this time and were not yet clinically identifiable.

In another analysis of the Swedish registry data (2005 through 2009), Landfeldt & Strom (2012) reported that there was no difference between branded and generic alendronate when the rates of GI adverse events were assessed within the first six months of alendronate prescription. Similar to the study above, six months of observation may have been an insufficient period of time of observation to detect GI adverse events. Further study of additional large databases in this manner is warranted.

### Cost-effectiveness

Since generic alendronate has commanded such a large share of the osteoporosis treatment market in recent years largely owing to its low cost, numerous cost-effectiveness analyses have been conducted to compare the cost of preventing fractures with generic alendronate to other brand therapies, most often denosumab.

Nayak et al. (2012) used a Monte Carlo simulation model to assess when osteoporosis screening with appropriate treatment would become cost-effective in women 65 years of age or older. Analyses revealed that at prices below $200 per year, screening with appropriate alendronate treatment resulted in significant cost-savings (up to $343 per quality-adjusted life-year or QALY). While costs for alendronate between US $400 and $800 we no longer cost-saving, they were still cost-effective with incremental cost-effectiveness ratios (ICERs) from US $714 to $13,902 per QALY gained.

In a comparison of the cost-effectiveness of denosumab to bisphosphonates for the prevention of hip fracture, generic alendronate was used as the low-cost comparator in postmenopausal women with varying fracture risk (Parthan et al.^2013^). In the general analysis of cost-effectiveness with the overall postmenopausal population, the use of denosumab was cost-effective, even when compared to generic alendronate (ICER $US 85,100/QALY). In the high risk subgroup consisting of women with two or more risk factors for fracture, denosumab was comparable to generic alendronate with an ICER of $US 7,900/QALY. Further, when the analysis was extended to women 75 years of age or older, denosumab was superior to generic alendronate and all other comparators. In an analysis using a Markov simulation model, denosumab was found to be a cost-effective replacement for generic alendronate when the 10-year risk of major osteoporotic fracture was 32% or greater (Strom et al.^2013^). Another Markov model simulation of the cost-effectiveness of denosumab modelled on Swedish data was completed by Jonsson et al. (2011). Treatment with denosumab was more expensive initially than all other therapies investigated, substantially more than generic alendronate. In this model, the ICER for denosumab as compared to generic alendronate was about Euro 27,000, but the marked discontinuation rates associated with generic alendronate use offset many of the gains of generic alendronate being low-cost. A similar investigation from Belgium concluded that denosumab was cost-effective when compared to other bisphosphonates, including generic alendronate, for the treatment of postmenopausal osteoporosis in women 60 years of age or older with an osteoporotic bone mineral density or with a prevalent vertebral fracture over a three year period (Hiligsmann & Reginster^2011^). One assumption made in this analysis, however, was that the persistence to denosumab would be 46% higher than to the oral bisphosphonates.

The cost-effectiveness studies have shown that generic alendronate, while being the least expensive alternative for the treatment of osteoporosis, can often be supplanted by other therapies as a first choice when long-term costs are considered, particularly when assessing the high rates of discontinuation assumed for generic alendronate in many of these models.

Of note, in many countries the manufacturer of brand alendronate substantially lowered their pricing of brand alendronate, now combined with vitamin D, to be similar to that of generic alendronate. This relatively recent change requires for new cost-effectiveness studies to be completed with the newer formulation of alendronate and vitamin D with the new pricing.

## Conclusion

Alendronate continues to play an important role in the management of osteoporosis. The proliferation of generic versions of brand alendronate has been rapid worldwide. Brand alendronate has undergone years of carefully monitored randomized placebo-controlled clinical trials, utilizing tens of thousands of osteoporosis patients, to establish tolerability, safety and efficacy. Further, numerous Phase IV investigations have supported the findings reported in Phase III clinical trials. The generic versions of alendronate have not been tested with the same rigour for tolerance, safety and efficacy.

Differences in excipients may be an important factor in the frequently reported differences in disintegration time between generic and brand alendronate. Most commonly, generic versions disintegrate far more rapidly than brand alendronate, increasing the potential for AEs. Differences in rupture characteristics of generic and brand alendronate may also increase the probability of oesophageal damage with the generic versions of alendronate.

Lastly, clinical data has consistently demonstrated that tolerance and effectiveness of generic alendronate is lower than that of the brand versions.

While generic substitutions may lead to equivalent outcomes to the brand formulation in other drug classes the weight of evidence would suggest this is not the case with alendronate; the efficacy, effectiveness and tolerance ascribed to brand alendronate should not be extrapolated to the relatively untested generic versions of alendronate.

## Authors’ original submitted files for images

Below are the links to the authors’ original submitted files for images. Authors’ original file for figure 1
