# Supplementary figures and images for: A critical review of brand and generic alendronate for the treatment of osteoporosis
Source: Springerplus. 2013 Oct 21;2(1):550. doi: 10.1186/2193-1801-2-550 (PMC4320211; doi:10.1186/2193-1801-2-550)

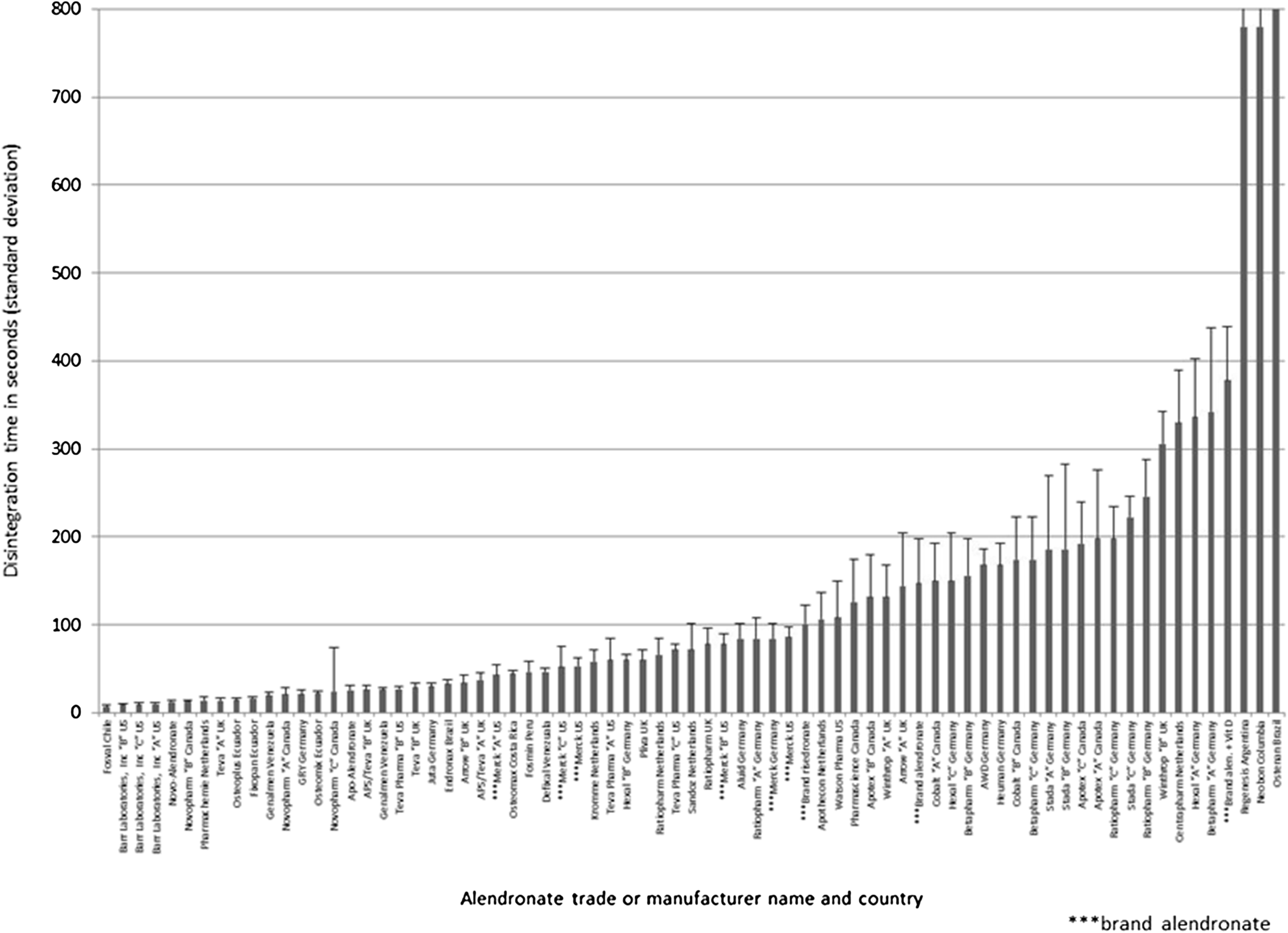

Supplement: Supplementary file 1 — Authors’ original file for figure 1 [file 40064_2013_1426_MOESM1_ESM.tif]
